# Mr. Buchanan on the Anatomy and Diseases of the Ear

**Published:** 1824-06-01

**Authors:** 


*An Engraved Representation of the Anatomy of the Human Ear, exhibiting, in one view, the External and Internal Parts of that Organ, in situ. Accompanied with a Plate of Outlines and References, with copious Explanations. To which are added, Surgical Remarks, &c. and a Synoptical Table of the Diseases of the Ear: the whole designed as a Guide to Acoustic Surgery.* By Thomas Buchanan, C.M. Licentiate of the University of Glasgow, &c. Hull, 1823, 12s. 6d. boards.

Mr. Buchanan thinks that few, if any, of the many plates of the human ear, that have been published, give a correct representation of the relative situation of the parts. Those of the late Mr. Saunders are acknowledged to be the best; "but even these shew only parts of the organ detached."

"Aware of the great defect in the best plates of the ear, and to form a model for my future dissection, I cut a cranium so as to admit of an oblique view from the temple to the tympanum; and with great care and perseverance was enabled to show the whole of the organ, both externally and internally, from this aspect, and in connexion with the surrounding parts." *Introduction.*

This attained, our author procured the head of an adult male, and after injection, he proceeded to form a preparation on the model of the cranium, from which, when finished, the drawing here represented was taken.

The first part of the work, containing three chapters, is dedicated to pure descriptive anatomy—and this we must, of course, pass over, with the remark that to us the descriptions appear to be correct. The second part of the volume, containing two chapters, contain surgical remarks on the introduction of the probe and catheter into the eustachian tube by the nostril—and on the operation of puncturing the membrana tympani. To these we shall dedicate a few pages of our journal.

Cap. I.—Before a young surgeon attempts the introduction of a probe or catheter through the nostril or eustachian tube, Mr. B. advises him to make the experiment first on the dried preparation, and then, if possible, on the recent subject. When the probe is just entering the mouth of the tube, (on the dead subject) the surgeon should mark it slightly in a line with the tip of the nose, and from this mark measure off the length of the tube outwards, marking it also. These marks will be useful afterwards in operations on the living body. Probes should also be kept ready curved and marked—some for the right, some for the left ear, being of various assortments to suit the size of the patient's head.

"The great difficulty of introducing the probe into the eustachian tube through the nostril, is the excessive irritability of the Schneiderian membrane with which it is lined.

"To overcome the disagreeable sensation produced by the introduction of foreign substances, it might be advisable to inject tepid water into the nostril, and at the same time dip the probe into ol. amygd. warmed to the temperature of the blood.

"Having got the patient seated conveniently, introduce the probe into the nostril, and when you approach with the first mark towards the tip of the nose, cautiously endeavour to find the mouth of the tube, by directing the knob on the end of the probe outwards, and rather upwards, until it has entered the labia.

"Having entered the tube, observe the distance of the second mark, and proceed in the same cautious manner until it is in a line with the tip of the nose, and feeling no resistance made to the probe you may conclude that it has entered the tympanum.

"But beware of farther introduction; for by rashly pushing the probe into the tympanum, you might injure the mechanism of the ossicula auditus, which, besides putting your patient to great pain, would throw discredit on the operation.

"If you wish to inject tepid water into the tympanum, provide a slender silver catheter, which should not exceed in diameter from the point to the first mark, that of the diameter of the knob of the probe.

"It should gradually increase in size from the first mark, for about a quarter of an inch, in length, to the thickness of a crow quill, and then be of an equal diameter to the end. This equal portion of the catheter to be made with a male screw on the outside, to fit a round flat piece of silver with a female screw in its centre.

"This flat piece of silver, which has been called a frontlet, ought to have five or six holes in its edges, so that it may be secured in a proper position by means of a ribbon passed round the head and through two of the holes of the frontlet, the ends of the ribbon to be then brought backwards and tie behind.

"If the end of the catheter were made to fit the little silver syringe used in Fistula Lachrymalis, it would be both useful and economical.

"Introduce the catheter in the same manner as the probe, and when the point has entered about half an inch into the tube, screw on the frontlet close to the tip of the nose; fasten it with the ribbon, and then inject the water through it with the syringe. The water used ought to be distilled, and by this means the pus or mucus in the Tympanum, will be held in solution, and be more easily evacuated than when precipitation takes place. It ought likewise to be heated to 94° Fahrenheit, but if a thermometer cannot be conveniently got, dip the hand into the water, and its temperature may be easily judged by the sensation produced on the back of the hand.

"After the operation a saline purgative ought to be given, and if considerable irritation arise, venesection with antiphlogistic regimen to be prescribed; and the patient confined to his room until all symptoms of inflammation have been subdued." 31.

If, from some cause or other, the operation should not succeed, and there is reason to suppose that the closure of the tube is not permanent, but merely from tumefaction or stricture of the parts, arising from recent exposure to cold, the patient should be bled with leeches near the mastoid processes—take sulphate of magnesia in small doses, and afterwards be put on an alterative course, with blisters behind the ears. By perseverance in this course, the tumefaction will decrease—the probe may again be tried; and, if adhesion has not taken place to almost the length of the tube, it may, with care, be rendered pervious.

### CHAP. II.—On Puncturing the Membrana Tympani.

Our author supposes that the attempts to introduce the probe into the eustachian tube had repeatedly failed—that the patient is unable to inflate the membrana tympani—and remains deaf, or nearly so. The operation of puncturing the membrana tympani is then admissible.

"For this purpose the surgeon ought to have an instrument (which I have called a perforator) of the following description :—

"The blade to be about three inches in length, of a quadrangular shape for about a quarter of an inch near the point; the other parts of the blade round, and increasing a little in thickness.

"The sides of the quadrangular part to be ground equally and brought to a point; the shoulders of the edge, as they are called, to taper gradually, so as to be rather more than a line in length. The diameter of the quadrangular part should not exceed one fourth part of a line, but the rest of the blade, especially near its insertion into the handle, to be about two thirds of a line in diameter.

"The handle to be octagonal, and made of mahogany stained black; about four inches in length, one line and a half in diameter near the blade, and increasing in thickness to about one line and two-thirds in diameter at the end.

"If the blade were shorter the handle would require to be proportionably advanced towards the Meatus, which would almost block up the tube, or at least obscure the view of the Membrane, so that the surgeon would have to operate under considerable disadvantages: whereas, when the blade is of the above length, it will be in his power to observe the progress of the point of the perforator, and to act accordingly.

"A room with a window fronting the south should be chosen for the place of operation; and the patient placed on a low seat, so that the rays of the sun may fall into the Meatus.

"The Manubrium or handle of the Malleus will then be distinctly seen pointing downwards and inwards; occupying the superior half of the Membrana Tympani.

"The surgeon being seated on a high chair, should lay his left hand on the head of the patient,* and with the right take hold of the instrument in the same manner as he would a pen when writing; he should then, cautiously and steadily, enter the point of the perforator into the Membrana Tympani, about half-way between the centre and its lower edge, and with the thumb and index finger give the instrument half a turn one way, and then half a turn the other, and in this manner gently push the point about a line through the Membrane.

"The operation will be performed with greater precision if the sur-

---

* "The head of the patient ought likewise to be secured by an assistant."

geon bring the thumb and index finger of his left hand nearly over the Meatus, and slightly grasp the perforator about the middle of the blade; by this means the point of the instrument will be kept steady, that is to say, from describing a circular evolution during the rotatory motion necessary to *this mode* of puncturing the Membrana Tympani.*

After this operation, our author recommends a saline purgative; and, if painful sensations are felt in the parts, venesection should be performed, confining the patient to his room, and keeping him on the most antiphlogistic regimen until the inflammatory symptoms are subdued, and the parts restored to healthy action.

Our author observes, that the *mode* of operating, in these cases, has been considered of little consequence, it being thought sufficient to introduce a sharp-pointed instrument through the membrana tympani, taking care to avoid injuring the ossicula auctitus. Mr. B. remarks that, from time to time, the deafness returns, requiring another and another operation, till, often, the membrana tympani is injured for ever. On examining the structure of this part, we find it of an oval shape, and muscular —the fibres running in a peculiar direction—from the circumference to the centre.

"Hence it is, that when the operator thought he was *cutting*, he was only *separating* the fibres by the mechanical power of the instrument; and that the union which took place afterwards, was an effort of nature to restore the fibres to their original position and tone. To render this still more plain, let the muscle of a living animal be laid bare, and an incision made into it, in the direction of the fibres; and the parts afterwards left to nature.

"Adhesion will take place in almost every instance. But let a muscle in a similar situation be cut across, and the fibres will immediately *retract* to an extent corresponding to the size of the wound.

"The peculiarity of the mode of operation which I would recommend, is, to *drill* the perforation, and by means of the quadrangular point of the Perforator the fibres of the Membrana Tympani will be *cut across*—

* "If any objections are offered to this mode of operation, as being too complex and difficult to execute, owing to the unsteadiness of patients in general. The surgeon might then have a Perforator made of the same length and thickness as the above, but spear pointed similar to a lancet; the shoulders not to exceed $\frac{1}{40}$ of an inch in breadth; the handle ivory, with two or three black spots inserted, corresponding to the flat sides of the instrument. The operation might then be performed by a simple puncture, by the surgeon keeping the flat side of the Perforator opposite to the centre of the Membrana Tympani when he operates, and by this means the fibres will be cut across; but there will be more risk of the closure of the puncture than in the mode described above."

retraction take place— the wound assume an oval figure, and there will be less danger of a union of the parts taking place than in the common mode of operating by a simple puncture.*

" It is for this reason I have been so particular in describing the Perforator and the manner of using it, in order that an operation the mode of which has hitherto been accounted of little or no moment, but which is of the utmost importance to the Patient,—should be performed in a manner agreeable to the *true* principles of Surgery."

We have now given a full account of the operative part of the work before us, (with the exception of the synoptical table) as far as the typography is concerned. The plate must be examined in the original, which, we have no doubt, will meet a favourable reception among those concerned in aural practice, if not among the surgical profession at large.

* " I have in several cases introduced the Probe through the Nostril and Eustachian Tube into the Tympanum, in the manner described in Chap. I. and likewise Punctured the Membrana Tympani with a quadrangular Perforator similar to the above, with success. The patient belonged to the Hull Dispensary for Diseases of the Eye and Ear.

" I intend to lay the history of these cases before the public at some future opportunity."

